# Comet Sign (and Other) in *Pyemotes* Dermatitis

**DOI:** 10.3201/eid1503.081461

**Published:** 2009-03

**Authors:** Juan B. Bellido-Blasco, Alberto Arnedo-Pena, Francisca Valcuende

**Affiliations:** Centro de Salud Pública—Epidemiología, Castellón, Spain (J.B. Bellido-Blasco, A. Arnedo-Pena); Hospital de la Plana (Castellon)—Dermatología, Castellón (F. Valcuende)

**Keywords:** Pyemotes, dermatitis, comet sign, epidemiology, letter

**To the Editor:** Recently, Pascal Del Giudice et al. published an interesting article (*1*) about dermatitis in France caused by *Pyemotes ventricosus* in which they highlight the presence of the comet sign in a number of their patients. It is, they assert, a sign that because of its peculiarity could be useful for diagnosing this type of dermatitis in outbreaks and sporadic cases.

Some years ago, we studied 3 outbreaks (with >100 cases) of dermatitis caused by *P. ventricosus* parasitic mites in Castellón, Spain, produced by different infected materials (*2*). When we published the results, we concentrated on the epidemiologic characteristics and the discovery of the mite; perhaps we paid too little attention to the appearance of the lesions, of which we did not provide images. Nevertheless, we also observed the descriptions by Del Giudice et al., which we now show in the Figure. In 2 patients (Figure, panels A and B), the comet sign can be clearly assessed; the patients were 2 women who had had direct contact with the infected material against their legs. The other patient (Figure, panel C) displayed 56 macules with 1 pruritic central vesicle. We did not observe facial lesions on any of the case-patients (but we did observe lesions on the necks of some patients).

**Figure:**
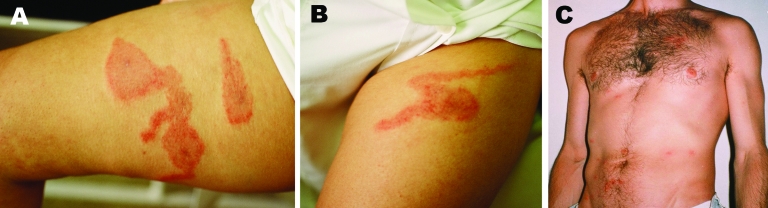
Photographs of 3 persons with skin lesions of *Pyemotes* dermatitis during the same outbreak in Castellón, Spain, showing the comet sign in 2 affected women (panels A, B), and macular form of the lesions in 1 of the affected investigators (panel C).

Our data coincided with those of the French study and reinforce the specificity of this dermatologic sign. However, this was not the only coincidence; cases also occurred among the investigators after contact with the infected material in each of the outbreaks. Perhaps both signs may characteristic this dermatitis: the comet sign and “the sign of the infected investigators” of the outbreaks.
